# Pyruvate is a prospective alkalizer to correct hypoxic lactic acidosis

**DOI:** 10.1186/s40779-018-0160-y

**Published:** 2018-04-26

**Authors:** Ying Wang, Ya Huang, Jing Yang, Fang-Qiang Zhou, Lian Zhao, Hong Zhou

**Affiliations:** 1 Institute of Transfusion Medicine, Academy of Military Medical Sciences, Beijing, 100850 China; 2 Department of Transfusion, Hainan Branch of PLA General Hospital, Sanya, 572013 Hainan China; 3 Fresenius Dialysis Centers at Chicago, Rolling Meadows Facility, Chicago, IL 60008 USA; 4 Shanghai Sandai Pharmaceutical R&D Co, Shanghai, 201203 China

**Keywords:** Type A lactic acidosis, Hyperlactatemia, Pyruvate, Glucose metabolism, PDH activator

## Abstract

Type A lactic acidosis resulted from hypoxic mitochondrial dysfunction is an independent predictor of mortality for critically ill patients. However, current therapeutic agents are still in shortage and can even be harmful. This paper reviewed data regarding lactic acidosis treatment and recommended that pyruvate might be a potential alkalizer to correct type A lactic acidosis in future clinical practice. Pyruvate is a key energy metabolic substrate and a pyruvate dehydrogenase (PDH) activator with several unique beneficial biological properties, including anti-oxidant and anti-inflammatory effects and the ability to activate the hypoxia-inducible factor-1 (HIF-1α) - erythropoietin (EPO) signal pathway. Pyruvate preserves glucose metabolism and cellular energetics better than bicarbonate, lactate, acetate and malate in the efficient correction of hypoxic lactic acidosis and shows few side effects. Therefore, application of pyruvate may be promising and safe as a novel therapeutic strategy in hypoxic lactic acidosis correction accompanied with multi-organ protection in critical care patients.

## Background

Lactic acidosis commonly occurs in numerous clinical conditions with severe pathogenic insults and indicates a serious prognosis. Lactic acidosis is referred to as acidosis with hyperlactatemia in terminology and is defined as metabolic acidosis with a serum lactate concentration > 4 or 5 mmol/L and an arterial pH (pHa) < 7.35. There are traditionally two categories of lactic acidosis: type A and type B. Type A lactic acidosis (LA) primarily results from hypoxic mitochondrial dysfunction, which causes lactate accumulation. It is a result associated with insufficient supply and underutilization of oxygen in tissues. In clinical settings, LA often appears as one of the severe complications in patients subjected to hypotension, as with hemorrhage, trauma and sepsis, and in patients with weakened oxygenation, such as with carbon monoxide, cyanide and iron poisoning, severe anemia, exhausted muscle activity (exercise, asthma) and hemoglobin-transfer disorders [1, 2]. Type B has a characteristic of hyperlactatemia, but clinical evidence of tissue hypoxia is absent. Type B is subdivided into B1 (associated with certain underlying diseases such as liver failure), B2 (due to drugs or toxins) and B3 (caused by inborn errors of metabolism) [2]. In the present paper, we mainly discussed the treatment of LA specifically referring to type A.

## Lactic acidosis (type A) in clinical settings

It has been long known that LA is an independent predictor of mortality for critically ill patients. A high lactate level is closely correlated with a poor prognosis in patients subjected to severe hemorrhage (HS). Abramson et al. [3] provided early evidence that trauma patients whose lactate levels were normalized (serum levels < 2 mmol/L) within the first 24 h in the intensive care unit (ICU) were more likely to survive, those who normalized between 24 and 48 h had a 25% mortality and those who normalized over 48 h after injury had a nearly 86% mortality. Furthermore, initial and peak lactate levels closely correlate with multiple organ dysfunction syndrome and survival rate following trauma [4]. Notably, relative hyperlactatemia in the normal reference range is also independently associated with increased hospital mortality in critical care patients [5]. Currently, the incidence of hyperlactatemia is over 30% and the mortality rate of LA is over 50% in children and adults in ICUs. The European guideline on the management of major bleeding and coagulopathy following trauma (fourth edition) [6] recommends serum lactate and/or base deficit measurements as sensitive tests to estimate and monitor the extent of bleeding and shock (Grade 1B). Early lactate-guided therapy is a beneficial method established for reducing sepsis mortality [7] and improving the outcome of critically ill patients [8]. However, current therapeutic agents are still in shortage and even somewhat harmful for LA treatment and the effort to discover effective agents remains necessary. In this paper, we reviewed data regarding LA treatment in animal experiments and clinical settings and strongly recommended that pyruvate as an optimal candidate might be a prospective alkalizer to correct LA in future clinical practice.

## Pathophysiological alterations of lactic acidosis

Type A LA is the direct result of cellular hypoxia. The main pathophysiological changes of LA are hypoxic metabolic disorders, which are mainly as follows.

### Inhibition of aerobic metabolism

During aerobic metabolism (oxidative phosphorylation), lactate and hydrogen ions (proton [H^+^]) are consumed in the mitochondrial respiratory chain to produce CO_2_, H_2_O and adenosine triphosphate (ATP). However, oxidation in mitochondria is significantly inhibited during cellular hypoxia due to depressed activity of a key enzyme, pyruvate dehydrogenase (PDH) complex and an insufficient oxygen supply. Therefore, lactate cannot be oxidized and [H^+^] is accumulated. Both are transported out of cells into the tissue interstitial fluid via the monocarboxylic acid transporter (MCT) and [Na^+^]/[H^+^] exchanger, resulting in hyperlactatemia and acidosis.

### Promotion of glycolysis

Hypoxia prompts anaerobic glucose metabolism, known as glycolysis, which produces two ATP molecules from one glucose molecule (2 glycolytic ATPs vs. 30 mitochondrial ATPs in aerobic metabolism). Endogenous pyruvate is converted by lactate dehydrogenase (LDH) into lactate, instead of into acetyl-CoA, in the glycolytic pathway. Prolonged and severe tissue hypoxia results in the generation of large quantities of lactate from anaerobic glycolysis and excessive [H^+^] from glycolytic ATP hydrolysis, or water dissociation according to Stewart theory. In addition, clinical use of adrenaline may also promote glycolysis to produce lactate.

### Reduction of lactate clearance/conversion

Lactate is produced mainly from skeletal muscle, skin, brain, intestines and erythrocytes [9]. Under pathological conditions, in which oxygen delivery is limited, lactate generation increases. A decreased lactate clearance is therefore an additional cause of increased lactate concentrations. Lactate is metabolized primarily in the liver and kidneys. During critical conditions, hypoxia often induces organ damage and gluconeogenesis via the Cori cycle may be finally inhibited due to dysfunction of the liver and kidneys, leading to a further decrease of lactate clearance.

## Current non-ideal therapeutic agents

### Alkaline agents

The administration of bases (alkalizers) is commonly regarded as the symptomatic treatment to correct metabolic acidosis, but it is unfavorable in LA corrections.

#### Sodium bicarbonate (SB)

Although it is not recommended in the majority of trauma guidelines, SB has been widely used to correct metabolic acidosis in clinical scenarios. Administration of SB can elevate the bicarbonate ([HCO_3_^−^]) level and raise the pH in plasma, which benefits patients with decreased serum [HCO_3_^−^]. However, SB may cause a series of complications. It increases carbon dioxide (CO_2_) production and decreases serum ionized calcium, which contributes to a decrease in ventricular and vascular contractility and damage to the myocardium. More importantly, SB therapy may induce an intracellular pH (pHi) decline. It was reported that bicarbonate administration led to an average increase in CO_2_ generation reflected by the need to increase ventilation by 40% to maintain a stable *p*CO_2_ [10]. For patients with abnormal respiratory compensation, CO_2_ generated from SB can rapidly diffuse into cytoplasm, which causes overproduction of intracellular [H^+^] [10]. The decreased pHi will worsen intracellular acidosis and damage tissues and organs. A randomized crossover study of 14 adults with lactic acidosis ([HCO_3_^−^] < 17 or base excess < − 10 and lactate nearly 7.8 mmol/L) treated with SB or equimolar sodium chloride demonstrated that SB treatment did not improve hemodynamics in ICU patients who suffered from LA [11]. It also cannot reduce but can even exacerbate the lactate accumulation [1]. Furthermore, SB has several negative side effects, such as altering blood pressure and triggering apoptosis [12]. SB administration requires a functional cardio-respiratory system to exhale the extra CO_2_ and there is a trend against using it in cardiac arrest patients. Therefore, SB use should be limited with caution to correct LA in critical care patients, i.e., avoiding the administration of SB if the pHa is > 7.10.

#### Tromethamine (THAM)

THAM is an amino alcohol with acid-buffering capacity [1]. The NH_2_ in THAM can bind [H^+^] to modulate both pHa and pHi as well as blood CO_2_ levels. However, it does not affect lactate accumulation. THAM also has toxic side effects such as respiratory depression and hypoglycemia [13]; thus, it is not suitable for LA treatment [1]. Moreover, except in a few case reports, THAM is no longer available for clinical use in most parts of the world.

### The activator of PDH

PDH is a key enzyme that modulates glucose oxidation, which converts pyruvate to acetyl-CoA. During hypoxia, PDH activity is significantly inhibited and then pyruvate is converted into lactate. Therefore, PDH activators enable correction of LA because of their ability to accelerate pyruvate oxidation and improve metabolic disturbances.

Dichloroacetate (DCA), the representative activator of PDH, has been administered since at least 1978 to patients with inborn errors of mitochondrial metabolism and is able to lower lactate concentrations and normalize blood pH. However, a large randomized controlled trial failed to demonstrate its effect on LA, illustrating that DCA increased arterial pH and decreased blood lactate but did not reduce mortality in ICU patients [14, 15]. A further study showed that DCA reduced mitochondrial NADH and elevated the incidence of premature ventricular contractions when glucose was the only exogenous fuel in isolated rat hearts during normoxic perfusion, which was mitigated by the addition of PDH substrates such as pyruvate [16]. Therefore, when using DCA to correct LA, PDH substrates may be needed. In addition, DCA may be harmful because it causes neuropathy [17, 18]. Nevertheless, the more rational treatment with appropriate doses of DCA to reduce side effects may need to be further investigated in patients with moderate or early-stage LA.

Other PDH activators, such as phenylbutyrate and desacylghrelin (DAG), are being studied [19, 20]. It was reported that phenylbutyrate increased the residual activity of PDH by increasing the proportion of unphosphorylated enzymes and had potential as a therapeutic agent for LA [21]. DAG, the precursor peptide of ghrelin, could normalize skeletal muscle lactate production and plasma lactate levels elevated by burn injury through the down-regulation of elevated inflammatory cytokines and activation of PDH [22]. However, the results were not ideal and further study is needed.

### Other chemicals

Many other agents are being investigated to better manage LA. For example, the compound 5-amino-2-hydroxymethylphenyl boronic acid, a phenyl boronic acid derivative, binds lactate and normalizes the blood pH by increasing the consumption of protons via the LDH pathway [12]. Spermidine, with its activating effect on PDH phosphatase, can also activate PDH, stimulating the decarboxylation of pyruvate and inhibiting lactate accumulation [1]. In addition, NHE1 (a cell-membrane [Na^+^]/[H^+^] exchanger) inhibitors may be useful in reverse of LA, but are still in animal studies [23]. Thus, these agents need more research to verify their effects and safety in patients.

## Anions in fluid therapy

Resuscitation to support circulation is one of the first steps in treating LA [22]. Intravenous (IV) fluids were first administered over 180 years ago [23] and fluid infusion is considered the mainstay of therapy for critical care patients. The organic acid anions in the fluids can be used as a source of base [22]. The effectiveness of various organic acids on LA correction is quite different, which is worthy of mention. Normal saline (NS) is often used as an initial resuscitation fluid in clinical settings. There is no doubt that NS is a reasonable alternative to restore perfusion if no other fluid is available. However, massive administration of NS often induces hyperchloremic acidosis [24, 25]. Recent studies suggest that NS may increase acidosis and the incidence of kidney injury in healthy volunteers or critically ill adults, mainly because of renal microvascular contraction induced by hyperchloremia [26, 27]. Therefore, the European guideline on management of major bleeding and coagulopathy following trauma (fourth edition) suggests that excessive use of NS be avoided (Grade 2C), although NS infusion with the potential to restore pH may be advantageous [6]. Metabolizable anions, such as lactate, acetate and malate, have been included in IV balanced solutions to avoid hyperchloremic acidosis. However, their effects in the treatment of LA vary and have not been compared rigorously. Here, we reviewed the composition of common fluids and their efficiency in LA correction in shock resuscitation.

### Lactated Ringer’s solution (LR)

The IV infusion of LR has been regarded as the standard regime and is widely used in the treatment of ICU patients, especially patients with acute massive hemorrhage. Yuan et al. [28] found that LR could alleviate brain trauma-precipitated LA in moderate-HS rats without the increase of arterial lactate levels, although a large amount of lactate was infused. Despite these advantages, some studies indicated LR-induced inflammation and hepatic apoptosis [29] and other studies even questioned its effect on acidosis correction [30]. Recently, it was demonstrated that LR infusion might be detrimental in resuscitation of severe HS in rats [31, 32]. In severe HS, lactate metabolism may be disturbed and the accumulated lactate can be further increased by LR infusion [32, 33], which aggravates the inhibition of glycolysis and affects organ function. The damaged liver and kidneys will further inhibit gluconeogenesis and cause lactate accumulation. Therefore, LR mainly affords plasma volume expansion, rather than improves acid-base disturbance. Although there is a lack of clinical evidence available, a large amount of LR infusion may exacerbate lactate accumulation in the resuscitation of severe or decompensated shock in ICU patients. In addition, the plasma lactate level is used as one of the diagnostic parameters in shock severity; thus, LR infusion may interfere with the diagnosis and treatment. Accordingly, the German guideline S3 for Patients with Severe and Multiple Injuries states that “the use of LR no longer appears to be worthy of recommendation”.

### Acetated Ringer’s solution (AR)

Historically, sodium acetate has been used as a fluid bath for hemodialysis [34]. Recently, AR, whose main component is sodium acetate, has been prevalently used in fluid therapy with both crystalloids and colloids in clinical resuscitation. It showed a significantly improved outcome (prolonged survival and less organ injury) in a rat model of severe HS compared with LR [33]. More importantly, AR showed a positive influence on the acid-base disturbance [35] because sodium acetate could alkalize plasma as quickly as SB. Acetate can be metabolized, via thiokinase consumption of 2 ATP molecules, to acetyl-CoA, which is later oxidized in the tricarboxylic acid (TCA) cycle. Acetate can also be converted to bicarbonate in the liver faster than lactate to raise the pHa [36, 37]. Furthermore, acetate is mainly degraded in the muscle, which is more ubiquitous than lactate degraded in the liver and kidneys. The metabolic velocity was the same rate as it was administered [38], even though the liver was damaged [39]. In addition, compared with LR, the administration of acetate-buffered solution did not show an elevated lactate concentration in Landrace pigs [40]. Therefore, the superiority of AR to LR is that AR does not inhibit glycolysis. However, impaired cardiac contractile response is a side effect of acetate if a large amount is infused [41]. Further, there was a case report of a surgical patient who first presented lactic acidosis from infusion of a higher load of sodium acetate (2 L/2 h), which likely delayed lactate clearance and inhibited the activity of PDH, the core enzyme of the TCA cycle [42]. Accordingly, AR is also not promising for correcting LA in critical care patients.

### Malate solution

Malic acid, in the form of its anion malate, is a trigger for the oxidation of acetyl-CoA and could increase the TCA metabolism [43]. Malate is a key anion in Jonosteril Malat (Fresenius Kabi) infusion, which is an appropriate primary fluid therapy for critical care in subjects with moderate and severe acidosis to maintain the perioperative fluid balance [44]. The intragastric administration of malate increased mitochondrial respiration and energy production in rats [45]. Resuscitation with malate also corrected LA in severe HS rats (MAP levels were maintained at 40 mmHg) [46]. However, malate cannot be metabolized through glycolysis and exhibits no protection of glycolysis under anaerobic metabolism and, thus, no red blood cell (RBC) protection. It may still not be optimal in the treatment of LA under fatal hypoxic conditions.

In all, current fluid therapies can correct LA to some degree, particularly in resuscitation of compensated shock, but the outcome depends on the anion selected in the fluids. The conclusion is that present IV solutions are not promising for LA correction in critically ill patients in ICUs. The pathogenesis of LA should be deeply understood to improve the clinical outcomes. As summarized above, activators of PDH play an important role in correcting LA because of their ability to improve metabolic disorders and decrease lactate accumulation, finally correcting severe acidosis.

## Pyruvate as a potential candidate for LA correction

The present agents for the treatment of LA are non-ideal. A therapeutic approach that can simultaneously correct acidosis and improve organ function with few adverse effects is of clinical importance. Pyruvate, a PDH activator and substrate, is capable of modulating blood acidic pH by improving metabolic pathways under hypoxia and thus may be an optimal agent in the correction of LA.

### Pyruvate and LA correction

Monocarboxylate pyruvate can effectively correct LA, an effect which has been demonstrated in many preliminary experiments. As early as 1999, Mongan et al. [47] first found that pyruvate resuscitation improved lactate metabolism and prolonged survival in a swine HS model. Later, the phenomena that pyruvate increased pHa and base excess (BE) and decreased lactate and the lactate/pyruvate ratio in swine [48] and rats [49] were found one after the other. Sharma et al. [50] showed that resuscitation with hypertonic sodium pyruvate elevated the blood pH up to nearly 7.42. Petrat et al. [51] studied the effect of pyruvate infusion in a model of rats subjected to severe mesenteric ischemia-reperfusion injury and found that pyruvate raised blood pH and BE. Koustova et al. [52] and Flaherty et al. [53] presented results that pyruvate Ringer’s solution reversed elevated blood lactate levels and preserved organ function in rats subjected to severe HS. Although the concept of pyruvate correction of LA was first proposed in 2004 [54], the conception was not demonstrated until the comparison of pyruvate Ringer’s solution with lactate Ringer’s solution in fluid resuscitation, in which pyruvate, instead of lactate, efficiently corrected hypoxic LA (increased pHa and BE, decreased blood lactate and the lactate/pyruvate ratio and doubled survival rates) in rats subjected to lethal HS in 2012 [55]. Although clinical data are not available, a case report of Leigh syndrome due to PDH mutation strongly suggests that oral pyruvate even corrects severe LA [56]. The entire reversal of LA by an administration of regular amount of pyruvate within hours in animal models implies its great clinical significance. The comparative effects of anions in fluid therapy on LA correction are listed in Table 1 [28, 31–33, 40–42, 44–49, 51–53, 57].

**Table 1:** The characteristics of different components in fluid therapy

Component in solution	Involved metabolic pathway	Effect on correcting LA	
		Advantage	Deficiency
Lactate	The end-product in the glycolysis pathway	Alleviating LA in moderate HS [28] through plasma expansion	Not working in severe HS and aggravating glycolysis inhibition and lactate accumulation [31–33]
Acetate	Metabolized to acetyl-CoA which is later used in the TCA cycle	Alkalizing plasma quickly, not inhibiting glycolysis and not elevating lactate concentration [40]	Side effect of impaired cardiac contractile response [41], inhibiting PDH activity [42]
Malate	The key intermediate in the TCA cycle	Working in cases of moderate acidosis [44] and moderate HS [46] and elevating energy production [45]	May not be suitable for the LA under severe hypoxic conditions
Pyruvate	The key intermediate in the glycolysis pathway and the TCA cycle	Correcting LA in moderate and lethal HS animals [47–49, 52, 53], isolated failing human myocardium [57] and ischemia-reperfusion injury model [51]	Not approved in clinic

### Pyruvate superiority in LA correction

The effect of pyruvate on LA correction is not solely a result of its chemical buffering ability as an alkalizer. Pyruvate has a low dissociation constant (pKa = 2.49), which represents its weaker buffering capacity relative to lactate (pKa = 3.9). Therefore, pyruvate may eliminate LA mainly via its biochemical characteristics in the promotion of energy metabolism and improvement of mitochondrial energetics to oxidize accumulated lactate and consume excess protons.

#### Improvement the cellular energetics and [H^+^] consumption

**Fig. 1:**
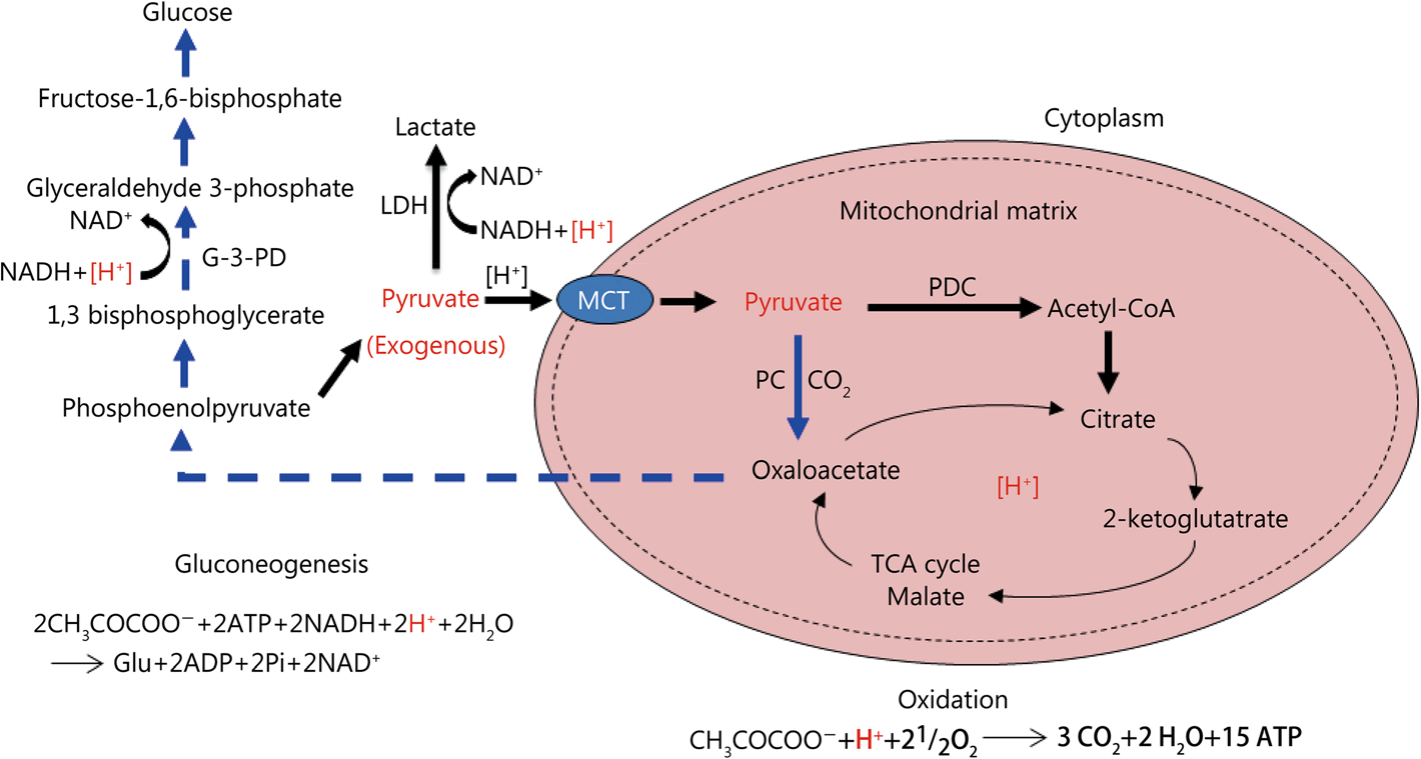
The metabolic pathways of exogenous pyruvate and their relationships to the consumption of [H^+^] First, pyruvate is oxidized into CO_2_ and H_2_O, which consumes an equal-molar [H^+^]. Second, when pyruvate is reduced to lactate, the process consumes an equal-molar [H^+^]. Finally, the transformation of pyruvate into glucose by gluconeogenesis consumes double-molar [H^+^]. [H^+^]: The cytosolic proton enters the mitochondria; [H+] (red): The proton being consumed in different glucose metabolic pathways; MCT. Monocarboxylate transporters; PDC. Pyruvate dehydrogenase complex; acetyl-CoA. Acetyl coenzyme A; PC. Pyruvate carboxylase; NAD^+^. Oxidized nicotinamide adenine dinucleotide; NADH. Reduced nicotinamide adenine dinucleotide; LDH. Lactic dehydrogenase; TCA. Tricarboxylic-acid.

Pyruvate is located in the metabolic center of three major substances in mammals, which connect glycolysis in the cytosol and oxidation in the mitochondria in glucose metabolism. It participates in several important metabolic pathways. The major acidosis-eliminating effect of exogenous pyruvate (Sodium Pyruvate, SP) can be illustrated in the following biochemical pathways (Fig. 1).

In the TCA cycle, pyruvate is converted into acetyl-CoA, catalyzed by the key enzyme PDH complex. Under hypoxic or ischemic conditions, PDH activity is inhibited. Notably, exogenous pyruvate can effectively restore the inhibited PDH activity through the decline of pyruvate dehydrogenase kinase activity [48] and can enhance the anaplerotic pathway (replenishment of TCA-cycle substrates) by improvement of pyruvate carboxylase (PC). Then, pyruvate is oxidized into CO_2_ and H_2_O in the TCA cycle, which consumes an equal-molar proton. Therefore, exogenous pyruvate prompts TCA cycle flux and accelerates lactate oxidation and [H^+^] consumption, raising the pHa and producing mitochondrial ATP.

It is reported that shifting the equilibrium of the LDH pathway towards the production of more lactate results in the consumption of protons and increase of the NAD^+^/NADH (nicotinamide adenine dinucleotide: oxidized form/reduced form) ratio via the hydrogenation of pyruvate [12]. Pyruvate is endogenously produced during glycolysis by breaking down glucose into two three-carbon molecules. Exogenous pyruvate, as a sole substrate, can be spontaneously converted into lactate by LDH, free from energy [55]. The pyruvate reductive reaction improves the NAD^+^/NADH ratio [55, 58], which is essential to sustain the glycolytic pathway and generate glycolytic ATP in anaerobic conditions. The reduction of pyruvate is a systemic alkalinizing reaction with [H^+^] consumption, raising the pHi in hypoxia and even anoxia [54, 59]. Pyruvate preservation of both the glycolytic pathway and LDH reduction is a unique superior property relative to other anions, including malate, in IV fluids. This beneficial attribute has recently been further argued by comparison of pyruvate-enriched oral rehydration solution (ORS) with citrate in ORS. Although both pyruvate and citrate in ORS have an equal buffering capacity equivalent to approximately 30 μmol of bicarbonate, only pyruvate in ORS can correct LA [60]. Neither bicarbonate nor citrate in ORS corrected hypoxic LA in animals subjected to severe shock.

Pyruvate protects multi-organ function in HS, including that of the liver and kidneys, the main organs for gluconeogenesis metabolism. Thus, exogenous pyruvate as a primary carbon source can enter into and prompt gluconeogenesis, generating glucose and consuming two [H^+^] [54, 61]. Furthermore, the improved function of heart, liver and kidneys resulting from exogenous pyruvate facilitates tissue oxygenation and lactate clearance.

In addition, pyruvate oxidative metabolism has the lowest oxygen consumption per ATP generation compared to other anions. Lactate oxidation to pyruvate by LDH requires approximately 0.5 ATP. Acetate oxidation generates 1/3 less ATP, and malate also produces much less ATP in mitochondria compared with equal-molar pyruvate.

Taken together, exogenous pyruvate correction of LA is primarily attributable to its key role in the improvement of glucose metabolism and cellular energetics in hypoxia. The underlying causes that facilitate pyruvate correction of LA may be closely associated with following factors.

##### Activation of HIF-1α

Several studies have demonstrated that pyruvate stabilizes hypoxia-inducible factor-1(HIF-1α) in normoxic conditions [62, 63]. HIF-1α is a key transcription factor that up-regulates a series of downstream genes involved in glucose metabolism, promoting the expression of glucose metabolism-related transporters and enzymes, such as glucose transporters [64], hexokinase [65], LDH-A [66] and phosphoglycerate kinase 1 [67]. The up-regulation of these proteins can increase the rates of aerobic glycolysis and enhance lactate oxidation and [H^+^] consumption. Moreover, erythropoietin (EPO), a typical up-regulative gene induced by HIF-1α, has cyto-protective and anti-inflammatory functions under various pathological conditions [68, 69]. It was demonstrated that pyruvate-enriched ORS (Pyr-ORS) significantly preserved the intestinal function and markedly elevated intestinal EPO in the enteral resuscitation of rats with burn injury compared with the effects of citrate-enriched ORS (Cit-ORS) [70]. Therefore, pyruvate may promote the correction of LA via its activation of the HIF-1α-EPO signaling pathway and improvement of cell metabolism.

##### Anti-oxidation / inflammation

Increased reactive oxygen species (ROS) lead to a cascade of events, including protein and fatty acid oxidation, and then suppress PDH activity during hemorrhage, trauma and sepsis. Pyruvate is a natural scavenger of oxidative/nitrosative stress. It directly reacts with free oxygen/nitrogen radicals, which generate in hypoxia, in a non-enzymatical and stochiometrical manner. It also exerts its antioxidant effects by increasing redox potentials in an indirect way [71, 72]. The anti-oxidative and anti-inflammatory effects may protect multi-organ function and, particularly, restore the inhibited PDH activity in hypoxia [49, 73], facilitating LA correction. Pyruvate is superior to the rest of the PDH activators mentioned above because it not only increases PDH activity but also provides PDH substrates in addition to its activation of HIF-1α and glycolytic protection.

##### Protection of red blood cell

The dual function of RBC is to deliver oxygen to tissues by 2, 3-diphosphoglycerate and to dilate the microvascular system as an oxygen sensor in hypoxic tissues by the release of glycolytic ATP. Moreover, RBC have a strong buffering capacity to sustain the acid-base balance via their O_2_- and CO_2_-transport ability. Pyruvate, in contrast with current anions in IV fluids, protects RBC function by preserving anaerobic glycolytic pathways. For example, preserving fluid containing SP could restore the oxygen-carrying capacity of stored RBC [74]. Pyruvate also protected RBC by sustaining ATP levels and inhibiting activated eNOS activity and NO production during an in vitro cardiopulmonary bypass procedure [75]. Therefore, pyruvate correction of LA may be additionally associated with the improvement of tissue hypoxia through RBC preservation. The above inference remains to be proven in shock resuscitation with pyruvate.

#### Variety of clinical routes of pyruvate administration

Pyruvate as a component in crystalloid solutions has been often reported [47–53, 57]. As a carrier solution in hydroxyethyl starch (HES) 130/0.4, it also showed a beneficial protection of the kidneys in animal resuscitation (J Surg Res. 2018; in press). Recently, various administration routes were studied. Yang et al. [59] reported that IV treatment with a small dose of sodium pyruvate ameliorated metabolic acidosis. Liu et al. [60] reported that Pyr-ORS fully reversed LA and markedly increased survival compared with the effects of Cit-ORS (WHO-ORS II) in dogs with severe burns. In addition, a very low dosage (< 0.2 g/kg) of Pyr-ORS was demonstrated to efficiently correct severe LA in rats with lethal HS in 4 h, doubling the survival rate [76]. A more recent investigation evidenced again that reduced osmolarity Pyr-ORS, instead of low-osmolar Cit-ORS (WHO-ORS III), effectively corrected LA and significantly elevated survival in rats subjected to lethal burn shock (J Surg Res. 2018; 225: 166-74.). In addition, pyruvate-enriched peritoneal dialysis solution, which was infused into the peritoneal cavity for adjuvant peritoneal resuscitation, was also shown to quickly correct severe acidosis in rats with HS [77].

On the other hand, in the past decade, numerous animal studies have demonstrated that ethyl pyruvate (EP), a derivative of pyruvate, protected multi-organ function with its preferable properties of anti-inflammation and anti-oxidative stress and inhibited elevated lactate in plasma and liver tissue in a septic model [78]. However, there is a distinct difference between EP and SP. The former is an ester, not a salt like the latter, from the chemical point of view. EP is neither a salt nor an alkalizer. EP has to be hydrolyzed via esterase or spontaneous hydrolysis to become pyruvate, resulting in [H^+^] accumulation. Therefore, EP, per se, could not correct metabolic acidosis, even though it reduced hyperlactatemia in rats subjected to septic shock [79].

## Conclusions

To sum up, efficient treatments for severe metabolic acidosis, particularly LA, are of clinical significance because of the severe adverse impacts of acidosis, including impact to cardiovascular function and cellular metabolism and systemic inflammatory reactions, producing a high mortality. Pyruvate is a key energy metabolic substrate and a PDH activator with several unique beneficial biological properties, including anti-oxidative and anti-inflammatory effects and the ability to activate the HIF-1α-EPO signal pathway. Exogenous pyruvate in sodium salt preserves glucose metabolism and cellular energetics superior to the effects of the anions bicarbonate, lactate, acetate and malate in IV fluids in the efficient correction of hypoxic LA by a regular dosage in lethal rodent models. Pyruvate-enriched IV solutions (both crystalloids and colloids) may be not only an agent of volume expansion but also a therapeutic agent for organ dysfunction and metabolic disturbance in clinical fluid resuscitation. Despite of the lack of clinical trials of LA correction by SP at present, a case report strongly indicated its high possibility [56]. Additionally, pyruvate has been applied in preliminary clinical trials through systemic injection of large doses and was shown to be effective and have few side effects [80, 81]. Pyruvate may be safe as a novel therapeutic strategy to correct hypoxic LA accompanied with multi-organ protection in critical care patients. Moreover, a severe concern regarding its instability in solutions has been preliminarily overcome with patents of stable pyruvate aqueous solutions in both China and the USA. Although data from animal and clinical trials with stable pyruvate solutions by the patented approaches are not available, an IV pyruvate injection from a powder preparation obtained prior to clinical use is currently available to correct LA in clinical trials, as demonstrated in animals in terms of its effectiveness and in humans in respect to its safety. It is likely that pyruvate-based IV solutions will become the third generation following NS and lactate/acetate Ringer’s solution in fluid therapy [55]. In addition, Pyr-ORS may be beneficial in peri-operative fluid management and pre-hospital rescue in a large scale, such as in earthquakes and terror attacks. Further studies on pyruvate correction of LA are urgently warranted.

## Abbreviations

AR: Acetated Ringer’s solution
ATP: Adenosine triphosphate
BE: Base excess
Cit-ORS: Citrate-enriched ORS
DAG: Desacylghrelin
DCA: Dichloroacetate
EP: Ethyl pyruvate
EPO: Erythropoietin
HS: Hemorrhagic shock
[H ]: Hydrogen ion
HIF-1α: Hypoxia-inducible factor-1α
ICU: Intensive care unit
IV: Intravenous
LA: Type A lactic acidosis
LDH: Lactate dehydrogenase
LR: Lactated Ringer’s solution
MCT: Monocarboxylic acid transporter
NAD^+^/NADH: Nicotinamide adenine dinucleotide: oxidized form/reduced form
NS: Normal saline
ORS: Oral rehydration solution
PDH: Pyruvate dehydrogenase
pHi: Intracellular pH
pHa: Arterial pH
Pyr-ORS: Pyruvate-enriched ORS
RBC: Red blood cell
SB: Sodium bicarbonate
SP: Sodium pyruvate
TCA cycle: Tricarboxylic acid cycle
THAM: Tromethamine.
